# Effects of Low Temperature on Pedicel Abscission and Auxin Synthesis Key Genes of Tomato

**DOI:** 10.3390/ijms24119186

**Published:** 2023-05-24

**Authors:** Sida Meng, Hengzuo Xiang, Xiaoru Yang, Yunzhu Ye, Leilei Han, Tao Xu, Yufeng Liu, Feng Wang, Changhua Tan, Mingfang Qi, Tianlai Li

**Affiliations:** 1 College of Horticulture, Shenyang Agricultural University, Shenyang 110866, China; mengsida@syau.edu.cn (S.M.); xianghengzuo8308@163.com (H.X.);; 2 Modern Protected Horticulture Engineering & Technology Center, Shenyang Agricultural University, Shenyang 110866, China; 3 National & Local Joint Engineering Research Center of Northern Horticultural Facilities Design & Application Technology (Liaoning), Shenyang 110866, China; 4 Key Laboratory of Protected Horticulture, Shenyang Agricultural University, Ministry of Education, Shenyang 110866, China

**Keywords:** tomato, low temperature, pollen development, abscission, auxin, auxin synthesis gene

## Abstract

Cold stress usually causes the abscission of floral organs and a decline in fruit setting rate, seriously reducing tomato yield. Auxin is one of the key hormones that affects the abscission of plant floral organs; the *YUCCA* (*YUC*) family is a key gene in the auxin biosynthesis pathway, but there are few research reports on the abscission of tomato flower organs. This experiment found that, under low temperature stress, the expression of auxin synthesis genes increased in stamens but decreased in pistils. Low temperature treatment decreased pollen vigor and pollen germination rate. Low night temperature reduced the tomato fruit setting rate and led to parthenocarpy, and the treatment effect was most obvious in the early stage of tomato pollen development. The abscission rate of tomato pTRV-*Slfzy3* and pTRV-*Slfzy5* silenced plants was higher than that of the control, which is the key auxin synthesis gene affecting the abscission rate. The expression of *Solyc07g043580* was down-regulated after low night temperature treatment. *Solyc07g043580* encodes the bHLH-type transcription factor *SlPIF4*. It has been reported that *PIF4* regulates the expression of auxin synthesis and synthesis genes, and is a key protein in the interaction between low temperature stress and light in regulating plant development.

## 1. Introduction

The plant hormone auxin plays a central role in shaping plant growth and development [1]. Auxin also plays a central role in control of organ abscission, and it is thought that changes in the auxin gradient across the abscission zone are the primary determinant of the onset of abscission [2]. After decades of genetic and biochemical studies, numerous core molecular components and their networks, underlying auxin biosynthesis, and transport and signaling have been identified [3,4]. The TAA/YUC pathway is the major endogenous auxin biosynthetic pathway involved in major biological processes mediated by auxin activity, and its conservation in the plant kingdom has been functionally examined in many plant species [5]. Many studies have shown that several *YUC* genes are highly expressed in seed tissues such as maize [6], rice [7], melon [8], and strawberry [9], suggesting that auxin biosynthesis via the TAA/YUC pathway may be dominant in flower and fruit development. *AUXIN RESPONSE FACTOR 17* (*AtARF17*) directly binds to the promoters of *CALS5* and *MYB108* to regulate pollen wall formation [10], and overexpression of *ARF17* in *Arabidopsis* leads to defects in tapetum development and male sterility [11]. *OsPID* regulates the transport of auxin by phosphorylating *PIN1*, changes the polar distribution of auxin, and regulates the production and development of rice floral organs; *OsPID* also participates in the regulation of rice floral organ development by interacting with transcription factors such as *OsMADS16* and *LAX1* [12]. *OsYUC11*-mediated auxin biosynthesis regulates rice endosperm development [13]. *OsFTIP7* interacts with *OsH1* and promotes its nuclear localization, directly represses the transcription of the auxin biosynthesis gene *OsYUCCA4*, and down-regulates auxin levels after stage 9, leading to up-regulation of JA levels required for anther dehiscence and pollen maturation [14]. Recent studies have excavated the homologous genes of *YUCCA* (*YUC*), a key rate-limiting enzyme for auxin synthesis in maize, and found that *ZmYUC2* and *ZmYUC4* are specifically expressed in the aerial root tips of maize and regulate the local synthesis of auxin in aerial root tips. This then affected the gravity and growth angle of aerial roots [15]. Elevated cytoplasmic levels of H_2_O_2_ caused a suppressed auxin signal in the early abscission stage and enhanced ETH production during abscission [16].

Tomatoes, an important fruit and vegetable in China, are often subjected to low-temperature stress during production, especially in protected cultivation. Low temperature can regulate the transport and signal transduction pathway of auxin [17], but there are few reports on the effect of low temperature on auxin biosynthesis [18]. Low temperature may reduce the auxin content in the ovary by causing stamen abortion, eventually leading to flower drop and fruit drop. Studies have shown that exogenous application of auxin can effectively rescue low-temperature-induced flower drop [19]. Studies have shown that low temperature affects auxin biosynthesis, transport, and signal transduction. The low temperature treatment of apple seedlings resulted in a significant decrease in auxin content in their roots [20]. Further transcriptome data confirmed that low temperatures suppress the expression of the auxin synthesis gene *YUC2* [21]. However, there are also results of increased auxin content at low temperature. For example, after wheat was treated at 4 °C for 21 days, the IAA content on the spikes increased significantly [22]. The effect of low temperature on auxin synthesis may have different effects due to differences in species and organs. YUC-dependent IAA biosynthesis plays a role in *Arabidopsis* seedling cotyledon somatic embryogenesis [23]. Recent studies on apical hook formation and maintenance found that cotyledon and shoot apical meristems are protected from mechanical damage during seedling emergence from soil, revealing an alternative auxin signaling mechanism [24]. Relatively low auxin levels on the convex side of the apical hook promote cell elongation, whereas higher auxin levels on the concave side [25] inhibit this process [26]. Higher KNOTTED1-LIKE HOMEOBOX PROTEIN1 (*SlKD1*) and FRUITFULL (*SlFUL2*) expression in the abscission zone (AZ), thereby perturbing the auxin response gradient and causing increased ethylene production, eventually lead to the initiation of abscission [27]. There have been new findings indicating that *SlHB15A* mediates the antagonistic effect of auxin and JA-Ile during tomato pedicel abscission, while auxin inhibits abscission through the SlHB15A-SlJAR1 module [28]. However, the involvement of YUC-mediated auxin in low-temperature flower and fruit drop is still unclear.

Tomato flower drop is mainly caused by biotic and abiotic stresses, with low temperature being the most common factor. Thus, studying the effect of mitigating or preventing low temperature on tomato flower drop has great practical value. Clarifying the relationship between low temperature, auxin, and tomato flower drop will provide a theoretical foundation for preventing low temperature from affecting tomato flower drop. In our previous study, through bioinformatics and qRT-PCR analysis, members of the auxin synthesis gene family *YUC* were found in the tomato genome and *SlFZY2*, *SlFZY3*, *SlFZY4-1*, *SlFZY5* were highly expressed in tomato floral organs; then, through field treatment experiments, the critical period of low temperature affecting tomato fruit setting rate was determined; virus-induced gene silencing (VIGS) technology was used to determine the effect of low night temperature on tomato flower organ abscission. The key genes of auxin synthesis, also through transcriptome sequencing, clearly show that low night temperature affects the expression of genes related to tomato stamen development. This study will lay the foundation for further analysis of the mechanism by which low temperature regulates auxin synthesis and affects tomato flower drop. Subsequently, it will provide a theoretical basis for preventing low temperature from affecting tomato flower drop.

## 2. Results

### 2.1. Effect of Day and Night Low Temperature Treatments on the Abscission Rate of Tomato Pedicel

**Figure 1:**
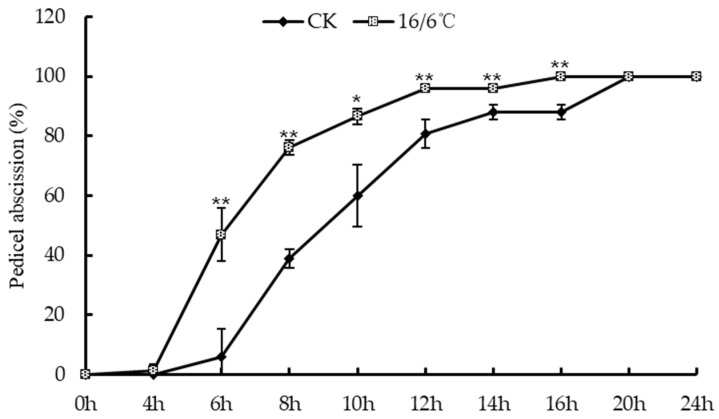
Peeling off rate of tomato petiole under low temperature treatment. * Indicates significant difference (*p* < 0.05, Student’s *t*-test), ** indicates extremely significant difference (*p* < 0.01, Student’s *t*-test).

The environment is an important factor leading to tomato flower and fruit drop, and low temperature is one of the environmental stresses that warm crops often suffer from. This study investigated the abscission rate of tomato flower stalks under low temperature stress day and night (Figure 1). The results are as follows: after being treated with low temperature 16/6 °C day and night, tomato flower stalks showed abscission at 4 h after flowering, and the abscission rate at 6 h was significantly higher than the control. The abscission rate at 16 h reached 100%; the tomato flower stalks in the control group fell off at 6 h and all fell off at 20 h. Research shows that low temperature treatment day and night promotes and accelerates the occurrence of tomato floral organ abscission.

### 2.2. Effect of Day and Night Low Temperature Treatments on the Vitality and Germination Rate of Tomato Pollen

**Figure 2:**
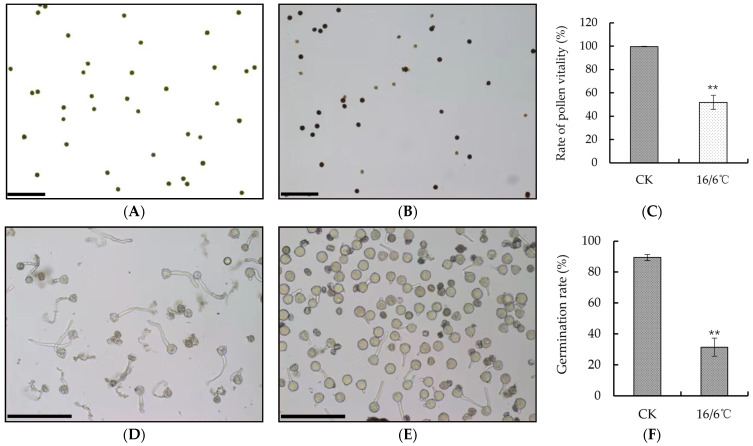
(**A**,**B**) The dyeing situation of tomato pollen vigor at 25/15 °C and 16/6 °C respectively, scale bar = 100 μm; (**C**) statistics of pollen vitality; (**D**,**E**) the pollen germination of tomato of 25/15 °C and 16/6 °C number, scale bar = 100 μm; (**F**) statistics of pollen germination rate. ** Indicates extremely significant difference (*p* < 0.01, Student’s *t*-test).

In this study, tomato pollen vitality and germination were observed under a laser confocal microscope using flowers that were fully opened under both day and night low temperature treatment and control (Figure 2A,B,D,E). I_2_-KI staining was used to observe the vitality of the tomato pollen. The pollen with deeper staining had higher vitality, while the pollen with lighter staining had lower vitality. After low temperature treatment at 16/6 °C, the pollen vitality was significantly lower than that of the control. Under low temperature, the pollen vitality was 51.84%, while the control was 99.67% (Figure 2C). Stamens were taken from low temperature and control flowering periods at day and night, the pollen was shaken out and added to the germination solution. After 0.5 h, the germination was observed under a microscope. The results showed that the pollen germination rate under low temperature was significantly lower than the control, with a pollen germination rate of only 31.34% under low temperature and 89.39% under the control (Figure 2F).

### 2.3. Effects of Day and Night Low Temperature on the Expression of Tomato Auxin Synthesis Genes

**Figure 3:**
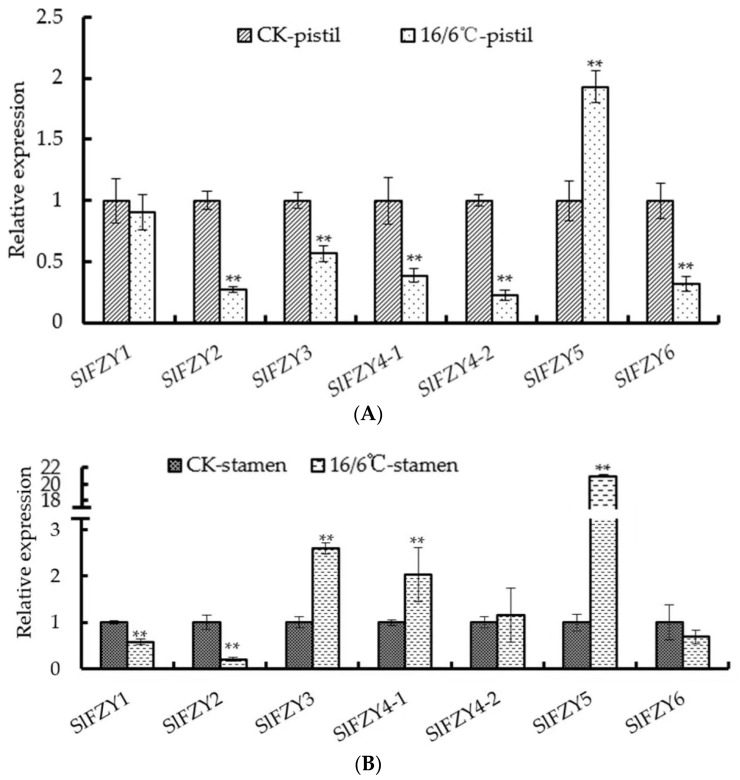
Analysis of expression levels of *SlFZY* in pistils (**A**) and stamens (**B**) in tomatoes under low temperature treatment. ** Indicates extremely significant difference (*p* < 0.01, Student’s *t*-test).

Auxin is one of the important hormones regulating organ shedding. In this study, in order to understand the mechanism of tomato pedicel shedding under diurnal low temperature, the expression level of the auxin synthesis gene *SlFZY* in pistils (Figure 3A) and stamens (Figure 3B) was analyzed after the tomato seedlings were subjected to low temperature. After diurnal low temperature treatment, the expression trends of different genes in different reproductive organs of tomato were not consistent. The expression levels of *SlFZY3* and *SlFZY4-1*, which were highly expressed in stamens, were up-regulated after low temperature treatment, but the expression levels in pistils were significantly lower than those of the control. *SlFZY1* and *SlFZY2* were opposite to these two genes, that is, they were lower in tomato stamens after 16/6 °C treatment, but higher in pistils. *SlFZY5* was significantly higher in tomato stamens and pistils than the control. The expression levels of *SlFZY4-2* and *SlFZY6* in tomato stamens had no significant difference between diurnal low temperature treatment and control, but in tomato pistil, the expression levels of these two genes were significantly lower than those of control after diurnal low temperature treatment.

### 2.4. Effect of Low Night Temperature on Fruit Setting Rate of Tomato

**Figure 4:**
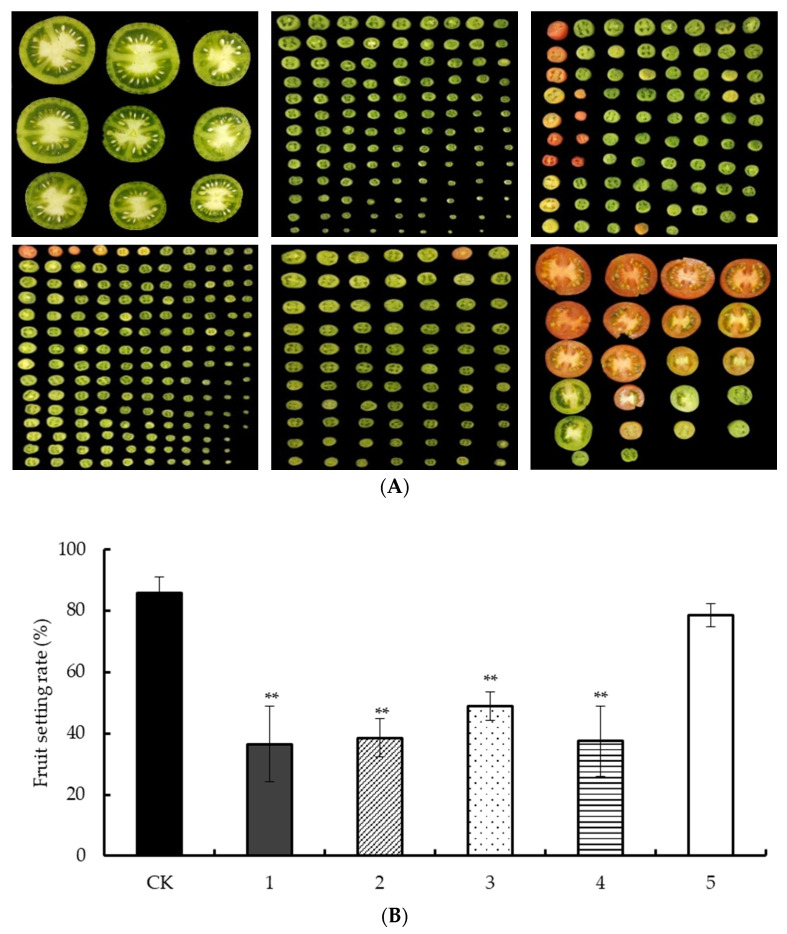
Investigation of tomato fruit set rate under low temperature treatment in different periods: in (**A**) from top to bottom, from left to right, they are CK, flower bud differentiation stage, stamen primordium differentiation stage, carpel formation stage, pollen development stage, and flowering stage, respectively; (**B**) uses CK, 1, 2, 3, 4, and 5 to represent, respectively. ** Indicates extremely significant difference (*p* < 0.01, Student’s *t*-test).

In this study, ‘AC’ tomato was used as the test material, and the low night temperature treatment of 25/6 °C was carried out in the artificial climate chamber at the early stage of flower bud differentiation, the stage of stamen primordium differentiation, the stage of carpel formation, the early stage of pollen development, and the flowering stage until the fruit setting stage. The statistical fruit setting rate is shown in Figure 4A. After investigation, it was found that the tomato fruit setting rate in the first four periods was significantly lower than that of the control, and the fruit setting rate in the fifth period was not significantly different from that of the control (Figure 4B). After the tomato fruit was cut crosswise, it was sent down. The low night temperature treatment led to parthenocarpy. The plants in the first four stages were all under low night temperature treatment from the early stage of pollen development to fruit setting, and there was no significant difference in fruit setting rate. Therefore, the early stage of selective pollen development is a critical period that affects the fruit setting rate after low temperature stress.

### 2.5. Effect of Low Night Temperature on Pollen Vitality of Tomato

**Figure 5:**
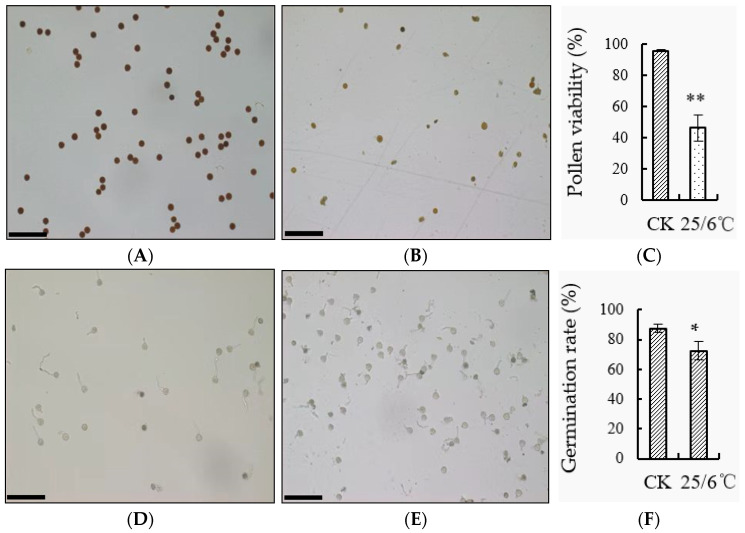
Effect of low night temperature on pollen vitality of tomato. (**A**,**B**) The dyeing situation of tomato pollen vigor under low temperature treatment at 25/15 °C and 25/6 °C, respectively, scale bar = 100 μm; (**C**) statistics of pollen viability; (**D**,**E**) the pollen germination of tomato under low temperature treatment at 25/15 °C and 25/6 °C, respectively, scale bar = 100 μm; (**F**) statistics of pollen germination rate. * Indicates significant difference (*p* < 0.05, Student’s *t*-test), ** indicates extremely significant difference (*p* < 0.01, Student’s *t*-test).

The pollen was taken from the tomato at flowering stage, and the pollen vigor and germination rate were counted under low night temperature treatment and control respectively (Figure 5A–F). The staining of tomato pollen I_2_-KI observed under the microscope after low temperature treatment was lighter (Figure 5A,B,D,E), and the statistics found that its viability was 46.24%, which was significantly lower than that of the control 95.74% (Figure 5C). The pollen germination rate of ‘AC’ tomato after low night temperature treatment was 72.28%, which was significantly lower than that of the control 87.32% (Figure 5F).

### 2.6. Investigation on Pedicel Abscission Rate of Silenced Tomato Plants with pTRV-SlFZY

**Figure 6:**
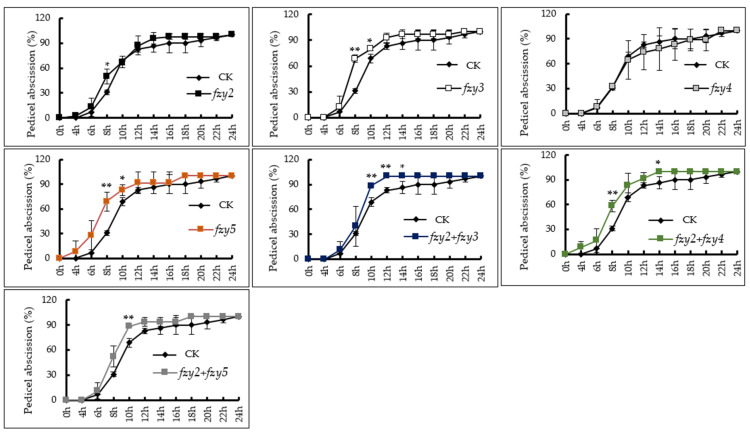
Petiole shedding rate of pTRV-*Slfzy* silent plants that were highly expressed in tomato flower organs. * Indicates significant difference (*p* < 0.05, Student’s *t*-test), ** indicates extremely significant difference (*p* < 0.01, Student’s *t*-test).

In order to preliminarily study the functions of the *SlFZY* family of genes found in tomato, this study used VIGS technology to silence four genes with high expression levels in floral organs and investigated tomato flower stalk abscission (Figure 6). The results showed that the flower organ abscission rate of pTRV-*Slfzy2* and pTRV-*Slfzy4* tomato silenced plants was not significantly different from that of the control. The abscission rate of floral organs of pTRV-*Slfzy3* and pTRV-*Slfzy5* silenced plants was extremely significantly higher than that of the control at 8 h after anthesis, significantly higher at 10 h, and higher than that of the control at 12 h to complete abscission. The tomato floral organ of pTRV-*Slfzy2fzy3* was significantly higher than that of the control at 10 h and 12 h after deflowering, and completely abscised after 14 h pTRV-*Slfzy2fzy4* tomato floral organs abscised at 4 h and completely abscised at 14 h, and the abscission rate at 8 h was 58.33%, which was significantly higher than that of the control (30.95%). The shedding rate of pTRV-*Slfzy2fzy5* at 10 h was 88.19%, which was extremely significantly higher than that of the control, at which point the shedding rate of the control was 68.81%.

### 2.7. Investigation on Pollen Viability of Silenced Tomato Plants with pTRV-SlFZY

**Figure 7:**
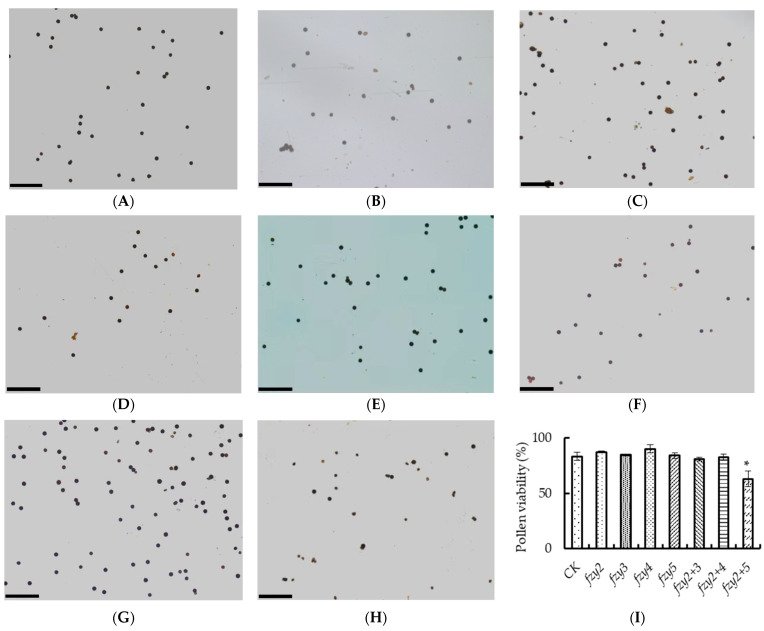
(**A**–**H**) pTRV-*Slfzy* silent plant tomato pollen vitality dyeing was observed by I_2_-KI staining. (**A**) Represents CK; (**B**–**H**) represent pTRV-*Slfzy2*, pTRV-*Slfzy3*, pTRV-*Slfzy4*, pTRV-*Slfzy5*, pTRV-*Slfzy2+3*, pTRV-*Slfzy2+4*, pTRV-*Slfzy2+5*, respectively, scale bar = 100 μm; (**I**) statistics of pollen vitality. * Indicates significant difference (*p* < 0.05, Student’s *t*-test).

I_2_-KI staining was used to observe the pollen viability of tomato pTRV-*Slfzy* silenced plants (Figure 7A–H). According to staining and statistics, only the pollen viability of pTRV-*Slfzy2fzy5* silenced plants was significantly lower than that of the control, and there was no significant difference between the other silenced plants and the control (Figure 7I).

### 2.8. High-Throughput Transcriptome Sequencing Analysis of Tomato Stamens under Low Night Temperature Treatment

Selecting an appropriate reference genome is an important step in information analysis. The sequence data clean data are compared with the reference genome, and the data comparison rate reflects the similarity between the sequenced sample and the selected reference genome. As shown in Table 1, the total mapped percentages of the three samples (CK1, CK2, CK3) under the control and the three samples (LT-1, LT-2, LT-3) under the low night temperature treatment are greater than 70%, multiple mapped is less than 10%, all of which meet the follow-up analysis requirements.

**Figure 8:**
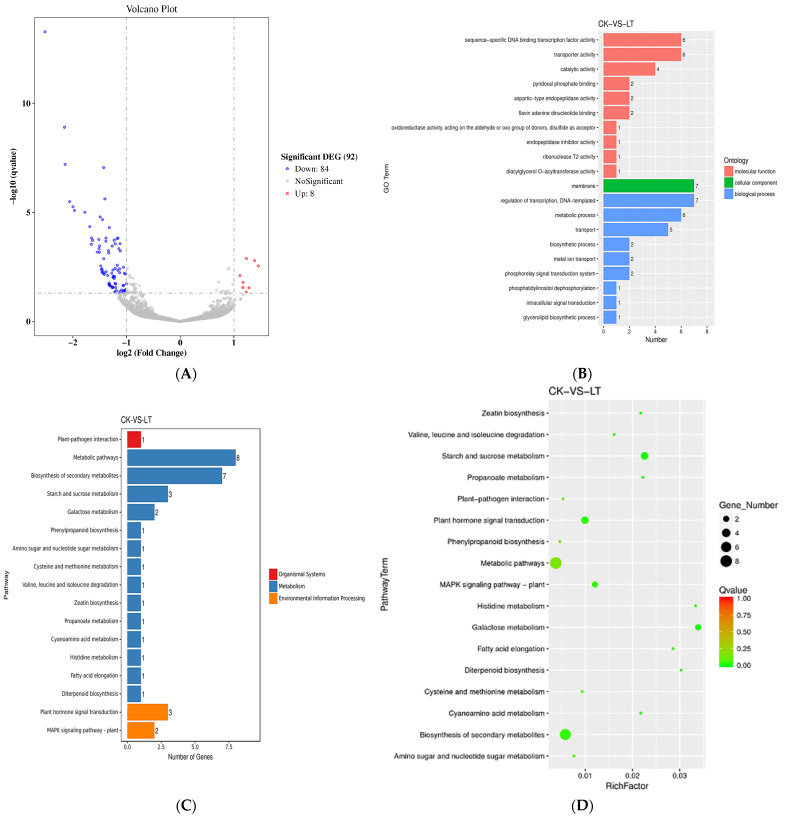
Transcriptome analysis of tomato stamens at low night temperature. (**A**) Differential gene volcano map; (**B**) GO enrichment histogram; (**C**) annotated histogram of significantly enriched KEGG annotation; (**D**) scattered graph of KEGG enrichment of differential genes.

In the volcano map of differential genes in tomato stamens under low night temperature treatment, when the q value of a gene is less than or equal to 0.05, and the expression changes by more than 2 times, the gene is considered to be a differential gene. If log_2_ (Fold Change) is less than −1, it means that the differential gene is significantly down-regulated, and if it is greater than 1, it means that the significant differential gene is up-regulated. Under the low night temperature treatment, there were 92 differential genes in tomato flowering stamens, of which 84 genes were down-regulated and 8 genes were up-regulated (Figure 8A).

The GO enrichment histogram of differential genes intuitively reflects the number distribution of molecular functions, cellular components, and biological processes. As shown in Figure 8B, in the GO enrichment of differential genes in tomato stamens under low night temperature treatment, 26 differential genes were enriched in molecular functions, 7 differential genes were enriched in cellular components, and 27 differential genes were enriched in set in biological processes.

In organisms, different genes participate in plant metabolism and signaling pathways through mutual cooperation, and through pathway significance enrichment analysis, the most important biochemical metabolic pathways and signal transduction pathways in which differentially expressed genes participate can be determined. KEGG enrichment analysis of differential genes in tomato stamens under low night temperature treatment found that there were five differential genes in environmental signaling pathways, of which three were involved in plant hormone signal transduction pathways and two were involved in plant MAPK pathways (Figure 8C,D).

Through KEGG analysis, we found that there were four down-regulated genes enriched in the plant hormone signal transduction pathway, namely *Solyc07g043580* encoding the bHLH-type transcription factor *SlPIF4*, the expression of *SlPIF4* was significantly down-regulated at low night temperature; the gene encoding *SlRRA7* protein *Solyc06g048600*; *Solyc06g048930* encoding TRR16-17 protein, SlRRA7 and TRR16-17 are induced by cytokinin in tomato root; *Solyc12g008900* encoding SlCKX6 protein, SlCKX6 is induced by cytokinin in tomato root (Table 2). *Solyc09g009110* encodes GA20 oxidase, which can catalyze the production of gibberellins with biological activity. MAPK3, encoded by *Solyc06g005170*, is a member of the tomato protein kinase gene family that plays an important role in mediating responses to biotic and abiotic stresses. SlWRKY33 encoded by *Solyc09g014990* is a member of the WRKY transcription factor gene family and has been related to multiple biological processes in plants. It has a C_2_H_2_ zinc finger domain and its expression level is down-regulated under low temperature treatment. *Solyc10g083290* encoded lin6, which is an extracellular invertase that catalyzes the cleavage of sucrose in the apoplast and its supply from the site of synthesis to the ‘sink’. Furthermore, lin6 is expressed in floral organs and roots, induced by glucose and zeatin. The 3-methyl-2-oxobutyrate dehydrogenase encoded by *Solyc06g05985* is a component of E1, and the expression of it and *Solyc09g009110* genes were up-regulated at low temperature (Table 2).

## 3. Discussion

Auxin is almost accompanied by the whole process of plant growth and development, and its locality is crucial to plant growth and development [29]. Low temperature stress is an important environmental stress that affects plant development, so it is speculated that there may be a regulatory relationship between low temperature stress and auxin. The transportation and signal transduction of auxin in tomato have been extensively studied; however, the research on its biosynthesis is still limited [30]. Under low temperature stress, the expression of auxin synthesis genes increased in stamens, but decreased in pistils. Low temperature treatment decreased pollen vigor and pollen germination rate. Low night temperature reduced the tomato fruit setting rate and led to parthenocarpy, and the treatment effect was most obvious in the early stage of tomato pollen development. The abscission rate of tomato pTRV-*Slfzy3* and pTRV-*Slfzy5* silenced plants was higher than that of the control, which is the key auxin synthesis gene affecting the abscission rate. The expression of *Solyc07g043580* was down-regulated after low night temperature treatment. *Solyc07g043580* encodes the bHLH-type transcription factor *SlPIF4*.

### 3.1. Low Temperature Promotes Abscission of Tomato Flower Organs

Auxin is locally synthesized [31,32,33], and analyzing the expression patterns of *SlFZYs* genes in different tissues in tomato is an important part of studying the function of this family of genes [34]. In our previous study, through qRT-PCR analysis, it was found that *SlFZY2*, *SlFZY3*, *SlFZY4-1*, *SlFZY5* were highly expressed in tomato floral organs, and it was preliminarily speculated that these four genes regulate the development of tomato floral organs. The expression level of *SlFZY3* was the highest in the underground part of tomato, the expression level of *SlFZY5* was the highest in the stem and leaf, and the expression level of *SlFZY4-2* was the highest in the young fruit.

Temperature stress includes high temperature and low temperature, which may be of reference to a certain extent [35]. At high temperature, *HISTONE DEACETYLASE 9* (*HDA9*) accumulates, facilitating the *H2A.Z* removal from the *YUC8* locus and providing a looser chromatin environment that allows *PIF4*-mediated activation of *YUC8* transcription [36]. High temperatures additionally promote auxin biosynthesis through the temperature-specific recruitment of *PIF4* to the promoters of the *IPyA* glycosylase *UGT76F1* and the IAOx-pathway-related *CYP79B2* gene to repress and promote their transcription, respectively, by unknown epigenetic mechanisms [37]. After the tomato was treated with diurnal low temperature during the flower bud stage, it was found that the diurnal low temperature promoted the abscission of tomato flower organs. Under diurnal low temperature stress, the expression of auxin synthesis genes increased in stamens, but decreased in pistils (Figure 3). The auxin in the tomato pedicel mainly comes from the base of the ovary, and the decrease of the auxin content in the ovary promotes the abscission of the pedicel. Diurnal low temperature treatment decreased pollen vigor and pollen germination rate (Figure 2). Stamen abortion is one of the important factors affecting flower and fruit drop. The increase of auxin content in rice stamens inhibits the synthesis of JA, which can lead to non-dehiscence of anthers and affect pollination [14]. The reduction of auxin content in pistil may be the result of poor pollination and fertilization.

### 3.2. The Early Stage of Pollen Development Is the Key Period When Low Night Temperature Affects the Fruit Setting Rate of Tomato

The traditional view is that auxin is mainly produced in vigorously divided apical meristems, young shoots, leaves, and germinating seeds, and is transported between cells through polar transport [18,38,39]. However, recent research advances have elucidated the molecular mechanism of auxin biosynthesis, revealing that its biosynthesis is not ubiquitous but localized [32,33]. Site-specific accumulation of auxin is regulated by the balance of cellular auxin efflux and local auxin biosynthesis [40]. The low temperature may affect the distribution of local auxin in the flower stalk, resulting in flower and fruit drop [41]. Low temperature is one of the main environmental factors affecting tomato yield, so this study screened the critical period when low night temperature affects tomato fruit set. Low night temperature reduced the tomato fruit setting rate and led to parthenocarpy, and the treatment effect was most obvious in the early stage of tomato pollen development, and the effect was less after tomato flowering, that is, after the pollen matured [42]. Through the detection of pollen viability and germination rate, it was found that low night temperature significantly reduced pollen viability and pollen germination rate. Low temperature at night leads to a decrease in fruit setting rate by affecting the development of pollen (Figure 4). Previous studies have shown that *Arabidopsis yuc2yuc6* mutants have reduced pollen vigor [43] and a lower seed setting rate than the wild type, indicating that auxin can regulate pollen development. It is preliminarily speculated that the increase of auxin content in stamens leads to decreased stamen fertility, developmental defects in unfertilized pistils, and the down-regulation of auxin synthesis gene expression levels, resulting in reduced transport of auxin to flower stalks, and eventually flower and fruit drop and parthenogenesis solid physiology.

### 3.3. SlFZY3, SlFZY5 Are the Main Auxin Synthesis Genes Affecting Flower Organ Abscission in Tomato

In this study, VIGS technology was used to verify the effect of auxin synthesis genes in tomato on abscission. The results found that pTRV-*Slfzy2* and pTRV-*Slfzy4* silenced plants had no significant difference in flower stalk abscission compared with wild type. After 8 h, the tomato pedicel shedding rate was significantly higher than that of the control, indicating that the gene family in tomato is functionally redundant. It is known that this family has been confirmed to have functional redundancy in *Arabidopsis* [31]. The conclusion of this experiment is consistent with that of the control group. The abscission rate of tomato pTRV-*Slfzy3* and pTRV-*Slfzy5* silenced plants was higher than that of the control. I_2_-KI staining showed that only when pTRV-*Slfzy2fzy5* was simultaneously silenced, the pollen viability of tomato was significantly lower than that of the control, which indicated that auxin synthesis deficiency in tomato affects pollen development (Figure 6).

### 3.4. Solyc07g043580 May Be a Key Transcription Factor Regulating Auxin Synthesis Genes in Tomato Stamens

High-throughput transcriptome sequencing was performed on the stamens of tomato at flowering stage after low night temperature treatment. A total of 94 differential genes were found under low night temperature treatment, of which 8 were up-regulated and 84 were down-regulated. GO enrichment analysis found that 26 differential genes were enriched in molecular functions, 7 differential genes were enriched in cellular components, and 27 genes were enriched in biological processes. Through KEGG analysis, it was found that three genes were enriched in the plant hormone signal transduction pathway, namely *Solyc07g043580*, *Solyc06g048600*, and *Solyc06g048930*, and these three genes were all down-regulated after low night temperature treatment. Among them, *Solyc07g043580* encodes the bHLH-type transcription factor *SlPIF4*. CBFs directly interact with *PIF3*, stabilizing the PIF3-phyB structure; on the one hand, inhibiting the expression of *PIF1*, *PIF4*, *PIF5*, and on the other hand, promoting the binding of ubiquitin ligase E3 to PIF1, PIF4, PIF5 protein and degrading it [44]. It is known that *AtPIF4* can activate the expression of *YUC4* in *Arabidopsis* [45], indicating that nighttime low temperature treatment in tomato stamens may affect auxin synthesis through the transcription factor encoded by *Solyc07g043580*, and low temperature stress and light play a role in regulating plant development. There are interactions between them. GO analysis showed that 2 genes were enriched in the MAPK signaling pathway, namely *Solyc06g005170* and *Solyc09g014990*. These 2 genes encoded *SlMAPK3* and *SlWRKY33* respectively, and their expression levels were both down-regulated. The *MAPK* family is involved in plant response to abiotic stress, and it is speculated that low night temperature can affect auxin synthesis by regulating *SlMAPK*. *MPK14*, another auxin-activatable *MPK* family member, was reported to phosphorylate and enhance the stability of non-canonical Aux/IAA Aux/IAA33 in root tips with high auxin concentrations [46]. *MPK1/2/14* were recently found to be activated by 1-naphthylacetic acid (NAA), suggesting a role for *MPK* family members in auxin signaling [47].

Low temperature may affect the expression of auxin synthesis genes through one or more transcription factors regulated by it, but it does not rule out the regulation of the expression of the *FZYs* family through epigenetics. It is speculated that there is functional redundancy in the tomato *FZY* family through VIGS, and further experiments are needed to verify the gene function. The effect of low temperature on the auxin content in tomato pistil may be through two ways: one is that low temperature regulates one or more transcription factors, which affects the expression of auxin synthesis genes in pistil, the other is that fertilization fails and auxin synthesis is reduced in the ovary. Low temperature and failure of pollination and fertilization may regulate auxin synthesis in tomato pistil through two different signaling pathways. Previous studies have shown that low temperature inhibits the transport of auxin, and low temperature may regulate the abscission of tomato floral organs by affecting the transport and signal transduction of auxin. Therefore, the research on auxin synthesis in tomato still needs to be further in-depth.

## 4. Materials and Methods

### 4.1. Plants and Growth Conditions

The tomato varieties tested in this experiment were ‘MicroTom’ and ‘Alisa Craig’ (AC). The ‘MicroTom’ was sown in the hole tray of the energy-saving solar greenhouse of the Facility Vegetable Research Base of Shenyang Agricultural University. When the seedlings sprouted flower buds, they were moved to the light incubator for treatment. The day and night low temperature treatment was 16 °C during the day and 6 °C at night; 25 °C during the day and 15 °C at night. The photoperiod was 12/12 h; the humidity was 65%; the light intensity was 400 μmol·m^−2^·s^−1^ [48]. The ‘AC’ was sown in the plug trays of the energy-saving solar greenhouse of Shenyang Agricultural University Facility Vegetable Research Base, and the flower bud differentiation stage, stamen primordium differentiation stage, carpel formation stage, pollen development stage, and flowering stage were respectively moved into artificial light source climate chambers. Then, night temperature treatment was carried out; night low temperature treatment temperature was 6 °C, control night temperature was 15 °C, and daytime temperature was 25 °C. The photoperiod was 12/12 h; the humidity was 65%; the light intensity was 400 μmol·m^−2^·s^−1^.

### 4.2. Extraction and Detection of Total RNA from Different Tomato Tissues

Take tomato roots, stems, leaves, flower buds, fully open flowers, stamens, pistils, and young fruits under normal growth conditions, and use Kangwei Century RNA Extraction Kit (CWBIO, Cambridge, MA, USA) to extract total RNA for analysis of the expression of the tomato *FZY* gene family in different tissues model. Tomato stamens and pistils under day and night low temperature treatment and low night temperature treatment were taken to prepare for analyzing the effects of different low temperature treatments on the expression of tomato *FZY* gene. The total RNA concentration was measured using a microplate reader (Thermo Fisher Scientific Inc., Waltham, MA, USA) and its integrity was checked by agarose gel electrophoresis [49].

### 4.3. Preparation of cDNA from Different Tissues of Tomato

The extracted total RNA was reverse transcribed using a TAKARA reverse transcription kit (Takara, Kusatsu, Japan). Reaction system: mix 4 μL; RNA + ddH_2_O 16 μL. After the reverse transcription procedure, the cDNA template was stored in a −20 °C low-temperature refrigerator.

### 4.4. Investigation on Abscission Rate of Tomato Floral Organs

Configuration of 1% agar medium: weigh 5 g of agar, add it to 500 g of distilled water, heat it twice in a microwave oven for 2 min each time, and stir it with a glass rod until it is completely dissolved. Pour the culture medium into a disposable plastic petri dish, cool and solidify at room temperature, and seal it with parafilm for later use [50,51]. The flower stalks of ‘MicroTom’ tomato at the flowering stage under the control and day and night low temperature treatment were taken, and the pistil base was removed with a double-sided knife, and then the proximal end was inserted into 1% agar medium, and the tomato flower organ abscission rate was measured in vitro. Taking the moment of inserting into the medium after deflowering as 0 h, the abscission rate was measured after 4 h, 6 h, 8 h, 10 h, 12 h, 16 h, 20 h, and 24 h, respectively. The number of pedicels that finally fell off was taken as the total number, that is, the shedding rate at this time was 100%; the shedding rate at other times was the percentage of the number of pedicels that fell off at this time to the total number of shedding [52].

### 4.5. Tomato Pollen Viability Assay

Configuration of I_2_-KI staining solution: weigh 25.4 g of I_2_ and 16.6 g of KI into a volumetric flask, add 900 mL of distilled water, dissolve completely, and dilute to 1 L [53]. Then pour into a brown threaded bottle and store in darkened light. At 9:00 in the morning, the flowers that fully opened under the control and low temperature treatment were taken, and the stamens were cut out with tweezers, cut into three sections with a double-sided blade, put into a 1.5 mL centrifuge tube, vortexed for five minutes, and the stamens were taken out with tweezers. Use a pipette gun to transfer 1 mL of staining solution into a centrifuge tube, then transfer the staining solution containing pollen into a cell culture dish and observe the staining situation with a laser microscope after 15 min [54].

### 4.6. Investigation on Pollen Germination Rate of Tomato

Weigh 2.62 g of MES into a volumetric flask, add 950 mL of sterilized water, and adjust its pH to 6.00 with NaOH. Take the tomato stamens under the control and low temperature treatment, shake out the pollen, add 1 mL pollen germination solution into the centrifuge tube with a pipette gun, then transfer the pollen germination solution containing pollen into the cell culture dish, and observe it with the laser microscope. After 30 min observe the germination [55].

### 4.7. Investigation on Tomato Fruit Setting Rate

‘Alisa Craig’ (AC) tomatoes treated with low temperature at night at different stages, when growing and developing to the fruiting stage, calculate the fruit setting rate of the first inflorescence.

### 4.8. RNA-Seq and Data Analysis

After low night temperature (25/6 °C) treatment at the early stage of tomato pollen development, the stamens at flowering stage were selected for high-throughput transcriptome sequencing. Total RNA was extracted using Trizol reagent (Invitrogen, Carlsbad, CA, USA), and mRNA was enriched with magnetic beads of Oligo (dT) for PCR amplification. Illumina Hiseq 4000 was used, followed by sequencing and bioinformation analysis in Suzhou Jinweizhi Biotechnology Co., LTD. After the library construction was completed, the library was diluted to 1.5 ng·μL^−1^ using Qubit2.0 Fluorometer for initial quantification, and the insert size of the library was tested using Agilent 2100 bioanalyzer (Agilent Technologies, Palo Alto, CA, USA). qRT-PCR was used to detect the library with a concentration higher than 2 nM, and Illumina sequencing was performed after qualified library detection.

The Gene properties were analyzed by the enrichment analysis method of Gene Ontology (GO), including molecular functions, cellular locations, and biological processes involved in the differential genes. Differentially expressed genes were selected with *p* < 0.05 correction value and significantly enriched on the GO pathway. KEGG was used to build the main public database for screening pathways. According to the data analysis of LT vs. CK, Rich factor was mainly included in the expression of enrichment degree.

### 4.9. Statistics Statement

Excel software was used for statistical analysis and mapping of differential genes and other data. SPSS24.0 software was used to calculate the mean and standard errors, and the significant differences were analyzed.

## 5. Conclusions

Tomato is one of the most widely cultivated heat-loving vegetables. However, in the north of China, low temperature stress often occurs during winter, particularly in protected cultivation. In this study, low temperature treatment was found to decrease pollen vigor and pollen germination rate. Low night temperature reduced the tomato fruit setting rate and led to parthenocarpy, and the treatment effect was most obvious in the early stage of tomato pollen development. The abscission rate of tomato pTRV-*Slfzy3* and pTRV-*Slfzy5* silenced plants was higher than that of the control, which is the key auxin synthesis gene affecting the abscission rate. The expression of *Solyc07g043580* was down-regulated after low night temperature treatment.

## Figures and Tables

**Table 1:** The match rate of RNA-seq data and reference genome.

Samples	Total Reads	Total Mapped	Multiple Mapped	Uniquely Mapped
CK1	43,826,382	42,216,609 (96.3269%)	1,540,385 (3.51474%)	40,676,224 (92.8122%)
CK2	45,355,570	43,806,526 (96.5847%)	1,696,148 (3.73967%)	42,110,378 (92.845%)
CK3	48,511,252	46,716,097 (96.2995%)	1,866,267 (3.84708%)	44,849,830 (92.4524%)
LT-1	51,842,542	49,771,809 (96.0057%)	2,092,856 (4.03695%)	47,678,953 (91.9688%)
LT-2	51,471,278	49,583,618 (96.3326%)	1,917,934 (3.72622%)	47,665,684 (92.6064%)
LT-3	44,275,212	42,862,271 (96.8087%)	1,619,842 (3.65858%)	41,242,429 (93.1502%)

**Table 2:** Differential genes.

Gene ID	log_2_ Fold Change	Regulation	Pathway
*Solyc07g043580.3*	−1.15946	Down	Plant hormone signal transduction
*Solyc06g048600.3*	−1.4375	Down	Plant hormone signal transduction
*Solyc06g048930.3*	−1.37844	Down	Plant hormone signal transduction
*Solyc01g079300.3*	−1.45545	Down	Galactose metabolism
*Solyc10g083290.4*	−1.31226	Down	Galactose metabolism
*Solyc07g063880.3*	−2.1405	Down	Biosynthesis of secondary metabolites
*Solyc02g085870.3*	−1.40118	Down	Biosynthesis of secondary metabolites
*Solyc02g091990.3*	−1.65493	Down	Biosynthesis of secondary metabolites
*Solyc12g011120.2*	−1.54504	Down	Biosynthesis of secondary metabolites
*Solyc08g068600.3*	−1.21645	Down	Biosynthesis of secondary metabolites
*Solyc06g059850.2*	1.381715	Up	Biosynthesis of secondary metabolites
*Solyc09g009110.3*	1.451812	Up	Biosynthesis of secondary metabolites
*Solyc06g005170.3*	−1.11532	Down	MAPK signaling pathway-plant
*Solyc09g014990.3*	−1.18645	Down	MAPK signaling pathway-plant
*Solyc12g008900.2*	−1.41223	Down	Zeatin biosynthesis
*Solyc07g063880.3*	−2.1405	Down	Starch and sucrose metabolism
*Solyc12g011120.2*	−1.54504	Down	Starch and sucrose metabolism
*Solyc10g083290.4*	−1.31226	Down	Starch and sucrose metabolism

## Data Availability

The authors will supply the relevant date in response to reasonable requests.
